# Cincho-Quinine

**Published:** 1877-07-20

**Authors:** Ferdinand King

**Affiliations:** Atlanta, Georgia


### SOUTHERN MEDICAL RECORD.

VOL. VII. ATLANTA, GA., JULY 20, 1877. NO. 7.

THOMAS S. POWELL, M.D.,

W. T. GOLDSMTIH, M. D.,

R. C. WORD, M. D.,

} EDITORS.

R. C. WORD, Business Manager.

SUBSCRIPTION: TWO DOLLARS PER ANNUM, IN ADVANCE.

☞ All communications, and letters on business connected with THE RECORD for 1877 must be addressed to the Business Manager.

### Original and Selected Articles.

### CINCHO-QUININE.

By FERDINAND KING, M.D., Ph. G., Atlanta, Georgia.

Modern medical economists have for sometime past been busily engaged in hunting a substitute for sulphate of quinine, in consequence of the scarcity and high price of that well known, valuable therapeutic agent. In their experiments and investigations, which have been more vigorously prosecuted in the Western and Southern States than elsewhere, they have not confined themselves exclusively to the small group of alkaloids derived from the cinchona, but they have tried almost everything possessed of a characteristic bitter taste, in which the anti-periodic or febrifuge properties of cinchona alkaloids might possibly be found. In searching for this longed-for substitute, our profession has been seriously imposed upon by an army of silver tongued agents, sent among us by unscrupulous, money-grabbing, Pharmacal manufactures, who, by their smiles and flattery, induce not a few of our most intelligent practitioners to try their quinine substitutes. In this way they create a temporary demand for their preparations, thereby enriching the new manufacturers at the expense of the physician's reputation, and oft-times the life of the unfortunate patient. As the effects of the cinchona alkaloids are sure and certain, I think we lose ground every time we deviate from the old and long beaten track. The scientific investigator has, so far, failed to find anything possessing even a tithe of their well-known anti-periodic and febrifuge properties. A new discovery, now and then, enjoys some reputation, but it is short lived, and soon succumbs to cinchona and its invincible allies.

There are, it is true, some serious objections to the employment of the alkaloids when we administer them *singly* or *uncombined*, such as urticaria, etc. These unpleasant symptoms, in a majority of instances, depend upon some idiosyncracy of the patient, just as catarrhal indications, not unfrequently follow the administration of iodide of potassium. These consequences, observable in such a small per cent. of patients where valuable therapeutic agents are administered, I am sure should not prejudice us against their general use. The same objections urged against the use of sulphate of quinine under certain pathological conditions may, with equal propriety, be applied, as above intimated, to the other single alkaloids of cinchona—i. e. quinidia, cinchonidia, quinquinicia and cinchonia. Unpleasant symptoms are noticeable when we administer any of the alkaloids, just enumerated, in large or heroic doses. To produce "quininism" by administering any of these single alkaloids, save quinine, we must employ larger doses than of the latter agent. For instance, let us take sulphate of cinchonidia. I think it perfectly worthless as anti-periodic, unless we give at least three times as much of it at a dose as of the sulphate of quinine. When given in large doses, we find all the symptoms of "quininism" present—such as cerebral disturbance, evinced by a feeling of tightness in the head, ringing in the ears, difficulty of hearing, etc. It seems that more or less of these symptoms must, in a measure, be secured before our patient can be relieved of his malarial depression ; therefore, as such a large quantity of sulphate of cinchonidia is required to procure the "quininism," I think we practice poor economy when we prescribe it.

The *modus operandi* by which cinchona or its alkaloids relieve malarial fevers, or influences, is not understood by a large number of our profession; and that it is often hypothetically employed by the average practitioner no one will pretend to deny. Many of these hypotheses are, no doubt, oftentimes correct, though the result of guess-work. We all know that the blood undergoes the most remarkable changes where patients are suffering from malarial poison. There is a marked increase in the quantitity of the plasma or alkaline fluid, while there is a corresponding decrease in the red globules or corpuscles. This condition is plainly evinced by the characteristic paleness always observable in patients suffering from malarial anæmia. Scientific and intelligent physicians will readily agree that it is necessary to restore the blood to its normal condition before we can bring back to our patient his wonted health. Therefore we should exame his blood, and learn what elements are wanting before we administer remedies. We should then give him such medicinal agents as are calculated to replace the lost elements and restore the blood to its original healthy condition. We find nothing in our materia medica that meets all the indications observed in patients suffering from malarial poison as completely as cinchona or Peruvian bark itself; but owing to its bulk, and the length of time required for its assimilation, it is objectionable. The ingenuity of the scientific and progressive pharmacist has, however, overcome these objections, and given us all the active principles of the Peruvian bark in a combination very appropriately called by its manufacturers (Messrs. Billings, Clapp & Co.,)

CINCHO-QUININE.

In this combination we find the nearest approach to the original substance, Peruvian bark, that modern science has yet attained. It is, as before stated, a combination of all the active medicinal principles of the best cinchona bark; and after the long and thorough test it has had, it stands unrivaled as a prompt, safe and uniformly reliable anti-periodic, possessing all the advantages and none of the disadvantages of sulphate of quinine, or any of the *single* alkaloids of the cinchona. It is entirely free from such external agents as sugar, liquorice, starch, magnesia, etc. It is wholly composed of the bark alkaloids, viz: quinia, cinchonia, quinidia, cinchonidia and the other alkaloidal principles which have not been distinctly isolated, and the precise nature of which are not well understood. Analyses attests the presence of all these alkaloids in cincho-quinine. In its preparation all the active tonic and febrefuge principles of the bark are secured without the bulky, inert, lignin, gum, tannin, etc. It exerts the full therapeutic influence of the sulphate of quinine in the *same dose*, without oppressing the stomach or creating nausea. It seldom produces cerebral distress, as quinine does, and I have found it to produce much less constitutional disturbance than the latter agent.

While engaged in the practice of medicine in the swamps of Alabama, where chills and fever were found in every family in my territory, during the late summer and early fall, I learned the value of cincho-quinine as a therapeutical agent in treating these maladies. I c uld not cure my patients with quinine alone, as it simply acted as a cerebral stimulant, and did not restore any of the lost elements to the blood. Having procured a sample bottle of cincho-quinine, and being pleased with its combination, I administered it to some of my *worst* cases, in whom I had the extreme pleasure of noticing a marked improvement from the very first dose, and a permanent cure at the expiration of one or two weeks. I found no difficulty in inducing the most delicate child, or squeamish female, to swallow the remedy, as it was quite soluble, almost tasteless, and did not leave that clinging, lasting bitter taste peculiar to the sulphate.

One of the main points I desire to impress upon the minds of those who read this article is, that cincho-quinine is not sulphate of quinine, it is not sulphate of cinchonidia, it is not cinchonia, but it is these and all the other alkaloids of cinchona in COMBINATION, and it is this composition, this representation of all the medicinal principles found in Peruvian bark that gives it the value claimed for it over and above all other preparations, or any one of the alkaloids of this valuable bark.

A majority of our oldest practitioners are agreed that larger quantities of quinine are required to treat, successfully, malarial diseases at the present time than when that salt was first introduced to the profession. This, in my opinion, is not owing, as many suppose, to the inferior quality of quinine as it is now found in the market, but because it is too *purely* and *solely* a sulphate, lacking in those properties that were found in the salt as prepared by pharmacists several decades ago. As produced then, it contained many of the alkaloids now found in cincho-quinine, and which contribute to the value of the latter combination as an anti-periodic and febrifuge.

The cincho is not "explosive" in its action, as in the *combination* the amount of nitrogen, always present in alkaloids, is greatly diminished by uniting them, hence less cerebral feelings and less constitutional disturbance follow its administration than are usually attendant upon the employment of the *single* or uncombined alkaloids. The same rules, as I before stated, are to be observed with regard to the use of the cincho-quinine that govern us when we employ the sulphate. It is given in the same doses, and where the *siege* treatment is indicated, it commends itself to the special attention of our profession everywhere.

Not only should the real value of cincho-quinine recommend that salt to physicians, but its low price is another inducement for us to prescribe it. It is less costly than the sulphate, while the dose is the same. The price of it fluctuates with the rise and fall of Peruvian bark, just as in the case of the sulphate, still it is at all times furnished at about half the cost of the latter article. Some may ask why cincho-quinine has not come into more general use in the South. To such I would say, it is from the fact that it has not been so extensively advertised as sulphate cinchonidia, chinoidine and some other anti-periodics of far less comparative value thet have poured into the offices of a large number of our physicians. The cincho-quinine stands on its own merits, and its use will be universal when it has been tried more thoroughly by the intelligent practitioner. I know personally, at this comparatively early period, of quite a number of physicians in the lower, or malarial regions, of our State who employ it to the almost entire exclusion of quinia and the single alkaloids of the same class. Indeed they use the latter only in purely nervous affections. It is not pushed upon our profession by an army of smiling agents that infest our country, popping up here and there, singing songs and tipping social glasses with the liberal members of our medical societies at their annual reunions or meetings. Its manufacturers claim no special praise for giving us this valuable anti-periodic and quinine substitute. And here I would state, rather parenthically, that "we are under lasting obligations" to no living firm of manufacturing chemists for any of the cinchona alkaloids, as they were all discovered prior to 1835. The praise for scientific discoveries should be given, in my humble opinion, to him who first makes it known, and not to those who grow rich from its profits.

